# Correction: Mortality and other outcomes of patients with coronavirus disease pneumonia admitted to the emergency department: A prospective observational Brazilian study

**DOI:** 10.1371/journal.pone.0248327

**Published:** 2021-03-04

**Authors:** Rodrigo A. Brandão Neto, Julio F. Marchini, Lucas O. Marino, Julio C. G. Alencar, Felippe Lazar Neto, Sabrina Ribeiro, Fernando S. Valente, Hassan Rahhal, Luz Marina Gomez Gomez, Caue G. Bueno, Carine C. Faria, Victor P. da Cunha, Eduardo Padrão, Irineu T. Velasco, Heraldo Possolo de Souza, Emergencia USP Covid group

The following information is missing from the Funding statement: This study was funded by Fundação de Amparo à Pesquisa do Estado de São Paulo (FAPESP) in the form of a grant awarded to ITV (2016/14566-4).

The seventh author’s name is incorrect. The correct name is: Fernando S. Valente.
