# Supplementary material for: Mortality and other outcomes of patients with coronavirus disease pneumonia admitted to the emergency department: A prospective observational Brazilian study
Source: PLoS One. 2021 Jan 7;16(1):e0244532. doi: 10.1371/journal.pone.0244532 (PMC7790269; doi:10.1371/journal.pone.0244532)
Supplement: Attachment — Submitted filename: Plosone_2Rev_Response_to_Reviewers.docx [file pone.0244532.s002.docx]

### Response to Reviewer Comments

We thank the reviewer and editors for their time and evaluation of our work. Following the valid critique of our shortcomings in the multivariate analysis we decided to start over our statistical analysis with Dr. Gómez who proposed we should use the least absolute shrinkage and selection operator (lasso) for multiple imputation and multivariate analysis. In the following section we present this change and a detailed point-by-point response to the comments. The original comments are provided in bold and our response immediately below.

**Additional comments from the editor:**

**Comment #N1**

1. Thank you for your response to our recent request regarding consent. To comply with PLOS ONE guidelines for human subjects research, please include the following information in the Methods section of your manuscript:

1) The reason why written informed patient consent was not obtained in some cases.
2) Whether the Research Ethics Committee approved the use of verbal authorization from the patient, and whether this authorization was informed.
3) Whether the Research Ethics Committee approved the use of verbal authorization from a family member when a patient was not able to consent.
4) How verbal consent was documented.

**Response #N1**

1) The research ethics committee approved verbal authorization when written informed consent was not possible. Written informed consent is not possible, for example, when the patient arrives in acute respiratory failure or is already unconscious due to sedation for mechanical ventilation. We have added this to the methods section of the manuscript.

2) Yes, the Research Ethics Committee approved the use of verbal authorization from the patient. We have added this information to the methods section of the manuscript.

3) Yes, the Research Ethics Committee approved the use of verbal authorization from the family member. We have added this information to the methods section of the manuscript.

4) The research ethics committee approved that verbal authorization should be documented in the patient's chart. We have added this information to the methods section of the manuscript.

**Original text:**

The study protocol was approved by the Research Ethics Committee (REC) of Hospital das Clínicas da Faculdade de Medicina da Universidade de São Paulo (protocol number CAAE 30417520.0.0000.0068) Patient anonymity was preserved. When written informed consent was not possible, verbal authorization from the patient or family members in the presence of witnesses.

**Revised text:**

The study protocol was approved by the Research Ethics Committee (REC) of Hospital das Clínicas da Faculdade de Medicina da Universidade de São Paulo (protocol number CAAE 30417520.0.0000.0068) with written informed consent or verbal authorization documented in the patient's charts. Patient anonymity was preserved. Written informed consent was not possible, for example, if the patient was unconscious or in acute respiratory failure. When written informed consent from the patient was not possible the REC approved informed verbal authorization from the patient or family members in the presence of witnesses.

**Comments from the editor:**

***Comment #1: Whether the Institutional Review Board approved the use of verbal authorization from the patient or family was obtained in the presence of witnesses. How verbal authorization from the patient or family was documented.***

***Response #1:*** In document #4.1119.252 from the HCFMUSP research ethics committee approved that verbal authorization can be obtained in the presence of witnessess and this should be documented in the patient's chart.

Comment #2: One of the noted authors is a group; Emergencia USP Covid group.

In addition to naming the author group, please list the individual authors and affiliations within this group in the acknowledgments section of your manuscript.

Please also indicate clearly a lead author for this group along with a contact email address.

***Response #2:*** *We have included this infomartion in the acknowledgements section and included more information on their affiliations*

Original version:

Emergencia USP Covid Group

Felipe Liger Moreira, MD1 , Edwin Albert D’Souza*1 , Arthur Petrillo Bellintani*1 , Rodrigo Cezar Miléo*1 , Rodrigo Werner Toccoli*1, Fernanda Máximo Fonseca e Silva*1, João Martelleto Baptista*1 , Marcelo de Oliveira Silva*1 , Giovanna Babikian Costa*1 , Rafael Berenguer Luna*1 , Henrique Tibucheski dos Santos*1 , Mariana Mendes Gonçalves Cimatti De Calasans*1, Marcelo Petrof Sanches*1 , Diego Juniti Takamune*1 , Luiza Boscolo*1 , Pedro Antonio Araújo Simões*1 , Manuela Cristina Adsuara Pandolfi*1 , Beatriz Larios Fantinatti*1 , Gabriel Travessini*1 , Matheus Finardi Lima de Faria*1 , Ligia Trombetta Lima*1 , Bianca Ruiz Nicolao*1 , Gabriel de Paula Maroni Escudeiro*1 , João Pedro Afonso Nascimento*1 , Everton Luis Santos Moreira*1 , Erika Thiemy Brito Miyaguchi *1 , Bruna Tolentino Caldeira*1 , Laura de Góes Campos*1 , Vitor Macedo Brito Medeiros*1 , Tales Cabral Monsalvarga*1 , Isabela Harumi Omori*1 , Diogo Visconti Guidotte*1 , Alexandre Lemos Bortolotto*1 , Rodrigo de Souza Abreu*1 , Nilo Arthur Bezerra Martins*1 , Carlos Eduardo Umehara Juck*1 , Felipe Mouzo Bortoleto, MD1 , Gabriel Martinez, MD1 , Lucas Gonçalves Dias Barreto, MD1 , Debora Lopes Emerenciano, MD1 , Daniel Rodrigues Ribeiro, MD1 , Danilo Dias de Francesco, MD1 , Eduardo Mariani Pires de Campos, MD1 , Stefany Franhan Barbosa de Souza, MD1 , Geovane Wiebelling da Silva, MD1 , Andrew Araujo Tavares, MD1 , Clara Carvalho de Alves Pereira, MD1 , Ademar Lima Simões, MD1 , Gustavo Biz Martins, MD1 , Maria Lorraine Silva de Rosa, MD1 , Thiago Areas Lisboa Netto, MD1 , Julio Cesar Leite Fortes, MD1 , Rafael Faria Pisciolaro, MD1 , Mauricio Ursoline do Nascimento, MD1 , Rodolfo Affonso Xavier, MD1 , Yago Henrique Padovan Chio, MD1 , Patricia Albuquerque de Moura, BS1 , Emily Cristine Oliveira Silva, BS1 , Ester Minã Gomes da Silva, BS1 , Yasmine Souza Filippo Fernandes, BS1 , Renata Kan Nishiaka, BS1 ,

* Medical Student

1Emergency Department, Hospital das Clínicas da Faculdade de Medicina da Universidade de São Paulo

Revised version:

Acknowledgements

The Emergencia USP Covid Group is composed by

Felipe Liger Moreira, MD**1 , Edwin Albert D’Souza*1 , Arthur Petrillo Bellintani*1 , Rodrigo Cezar Miléo*1 , Rodrigo Werner Toccoli*1, Fernanda Máximo Fonseca e Silva*1, João Martelleto Baptista*1 , Marcelo de Oliveira Silva*1 , Giovanna Babikian Costa*1 , Rafael Berenguer Luna*1 , Henrique Tibucheski dos Santos*1 , Mariana Mendes Gonçalves Cimatti De Calasans*1, Marcelo Petrof Sanches*1 , Diego Juniti Takamune*1 , Luiza Boscolo*1 , Pedro Antonio Araújo Simões*1 , Manuela Cristina Adsuara Pandolfi*1 , Beatriz Larios Fantinatti*1 , Gabriel Travessini*1 , Matheus Finardi Lima de Faria*1 , Ligia Trombetta Lima*1 , Bianca Ruiz Nicolao*1 , Gabriel de Paula Maroni Escudeiro*1 , João Pedro Afonso Nascimento*1 , Everton Luis Santos Moreira*1 , Erika Thiemy Brito Miyaguchi *1 , Bruna Tolentino Caldeira*1 , Laura de Góes Campos*1 , Vitor Macedo Brito Medeiros*1 , Tales Cabral Monsalvarga*1 , Isabela Harumi Omori*1 , Diogo Visconti Guidotte*1 , Alexandre Lemos Bortolotto*1 , Rodrigo de Souza Abreu*1 , Nilo Arthur Bezerra Martins*1 , Carlos Eduardo Umehara Juck*1 , Felipe Mouzo Bortoleto**, MD1 , Gabriel Martinez**, MD1 , Lucas Gonçalves Dias Barreto**, MD1 , Debora Lopes Emerenciano**, MD1 , Daniel Rodrigues Ribeiro**, MD1 , Danilo Dias de Francesco**, MD1 , Eduardo Mariani Pires de Campos**, MD1 , Stefany Franhan Barbosa de Souza**, MD1 , Geovane Wiebelling da Silva**, MD1 , Andrew Araujo Tavares**, MD1 , Clara Carvalho de Alves Pereira**, MD1 , Ademar Lima Simões**, MD1 , Gustavo Biz Martins**, MD1 , Maria Lorraine Silva de Rosa**, MD1 , Thiago Areas Lisboa Netto**, MD1 , Julio Cesar Leite Fortes**, MD1 , Rafael Faria Pisciolaro**, MD1 , Mauricio Ursoline do Nascimento**, MD1 , Rodolfo Affonso Xavier**, MD1 , Yago Henrique Padovan Chio**, MD1 , Patricia Albuquerque de Moura***, BS1 , Emily Cristine Oliveira Silva***, BS1 , Ester Minã Gomes da Silva***, BS1 , Yasmine Souza Filippo Fernandes***, BS1 , Renata Kan Nishiaka***, BS1 ,

* Medical Student

** Emergency medicine resident

*** Respiratory physiotherapy specialist

1Emergency Department, Hospital das Clínicas da Faculdade de Medicina da Universidade de São Paulo

Comment #3: We note you have included a table to which you do not refer in the text of your manuscript. Please ensure that you refer to Table 3 in your text; if accepted, production will need this reference to link the reader to the Table.

***Response #3:*** Thank for pointing out this out. We are now referencing Table 3 in the text.

Original text:

Of the 300 patients admitted to the ICU, 135 (45.0%) died, 146 (48.7%) were discharged, and 19 (6.3%) remained in the hospital.

Revised text:

Table 3 shows that of the 300 patients admitted to the ICU, 135 (45.0%) died, 146 (48.7%) were discharged, and 19 (6.3%) remained in the hospital.

Comment #4: Please include captions for your Supporting Information files at the end of your manuscript, and update any in-text citations to match accordingly. Please see our Supporting Information guidelines for more information: [http://journals.plos.org/plosone/s/supporting-information](about:blank)

Response #4: Our full database has been made available to PLOS ONE. I have translated any portuguese terms into english and reupdated the new version.

**Comments from the Reviewer:**

Comment #1: Has the statistical analysis been performed appropriately and rigorously? Reviewer #1: I Don't Know

Response #1:

Dear Dr. Steve Goodacre,

We would like to thank you very much for your honest opinion of our work. We have tried to release our work since August of the pandemic and we did not do the best job we could do in the statistical analysis work.

With some temporary loosening of COVID restrictions we were able to work with our staff statistician Dr. Gómez. She proposed we should use LASSO technique both for imputation and to derive which variables were independent and important for the outcome. We describe in the manuscript the technique we used. We believe it is much more robust and meaningful.

Comment #2: Please state the aim and specific objectives of the study at the end of the introduction, ensuring that the objectives are in accordance with the analysis undertaken (i.e. to describe the characteristics of people admitted to hospital with COVID-19 pneumonia and identify independent predictors of adverse outcome).

Response #2: Thank you for pointing this out. We added our objectives at the end of the introduction.

Original text:

In this study, we report our experience with these patients, all of whom were admitted owing to a confirmed or suggested diagnosis of COVID-19. All of them had pneumonia of at least moderate severity, which has been considered as a defining characteristic of COVID-19 by the WHO[5], and they all required hospitalisation.

Revised text:

In this study, we report our experience with these patients, all of whom were admitted owing to a confirmed or suggested diagnosis of COVID-19. Our objective is to report the characteristics of our patients, their clinical course during admission, their final outcome and finally to identify independent predictors of death. All of them had pneumonia of at least moderate severity, which has been considered as a defining characteristic of COVID-19 by the WHO[5], and they all required hospitalisation.

Comment #3: Under study design, it would be helpful to know the population covered by the hospital. Did the hospital act as the referral centre for the whole of Sao Paulo and, if so, what is the population of Sao Paulo?

Response #3: The university of São Paulo general hospital became and exclusive COVID center during the first wave of the pandemic. It was not the only referral covid centre but it was the hospital that received the most patients and the most severe patients. São Paulo city has a population of 12 million people but it is conurbated to various other cities and the greater São Paulo region has an estimated population of 21 million people.

Comment #4: Non-invasive ventilation is not reported in the outcomes. There have been reports that non-invasive ventilation (including CPAP) can reduce the need for ventilation and suggestions that this may improve outcomes. Are you able to comment on the use of non-invasive ventilation in your hospital?

Response #4: This is a really interesting question. In our current study, of the 506 patients with pneumonia on imaging, there were only 5 patients that received non-invasive ventilation. But, this was our early experience and we were following initial reports that non-invasive ventilation should be used sparingly.

Now, we also agree that non-invasive ventilation seems to reduce the need for mechanical ventilation. So currently we are using non-invasive ventilation much more commonly.

Comment #5: Please clarify the duration of follow up? Presumably patients were follow until death or hospital discharge, but a proportion were still in hospital at a specified date.

Response #5: The duration of follow-up was hospital discharge (alive or dead) or August 27 2020. We believe the percentage of patients that were still in hospital is small (3,9%). So as not to make even further delays in this revision we did not update the outcomes of these last 20 patients.

Comment #6: How were the 2219 ED attendances selected? I presume these were patients with suspected COVID-19, so how was that defined? I also assume that only those admitted were routinely tested for COVID-19, but please clarify this.

Response #6: These are the first 2219 patients that were evaluated in this service. Our service receives patients from other smaller services when the patient needs resources they do not have available (such as non-invasive ventilation, mechanical ventilation or ICU admission). It is uncommon for patients to enter directly into this hospital, but when they are unwell at our doorstep they are also admitted.

Normally we also admit patients from the specialty outpatient clinics when their physicians believe the patient needs to be seen in the ED. However, during the pandemic all outpatient clinics were closed.

Original text:

We found that 153 patients died (30.2%), 333 were discharged home (65.9%), and 20 (3.9%) remained in the hospital when this manuscript was finalised (Table 2).

Revised text:

We found that 153 patients died (30.2%), 333 were discharged home (65.9%), and 20 (3.9%) remained in the hospital when we finalised data collection on August 27, 2020. (Table 2).

Comment #7: It would be interesting to compare the characteristics and outcomes of those admitted with confirmed COVID-19 to those with negative COVID-19 testing. Maybe this is planned for a separate analysis.

Response #7: You are correct. We also believe it would be interesting to compare patients with negative COVID-19 testing. We are planning to write a second report with this data.

Comment #8: I recommend reporting numbers alongside the percentages in the results text.

Response #8: Thank you for this recommendation. We have added numbers as requested.

Original text:

The patients have had the symptoms for 8.5 days on average, before they were admitted to the ED, and 77.9% of patients had at least one comorbidity, of which the most prevalent was hypertension (55.3%), followed by diabetes (35.8%). Almost half of the patients had multiple comorbidities (49.2%). The most common symptoms were dyspnoea (76.1%), cough (74.3%), fever (45.7%), and myalgia (38.9%). In addition, 15.8% of patients had rhinorrhoea and 14.8% had sore throat. Anosmia and dysgeusia, symptoms that were common in other series, were also present in 23.1% and 19.6% of patients, respectively[1-3], while diarrhoea and nausea were present in 17.4% and 19.2% of patients.

Among the patients, 82.5% needed oxygen supplementation in the ED, 12.4% needed vasopressors immediately after arrival in the ED, and 8.7% were already intubated upon arrival to the ED.

Similar to other series, lymphopenia, defined as a lymphocyte count < 1500 cells/µL, was quite common (82.8%)[4, 5]. D-dimer levels were elevated in 83.5% of patients. Levels of lactate dehydrogenase, C-reactive protein, cardiac troponin T, and creatine phosphokinase were also elevated in 88.2%, 63.4%, 38.3%, and 27.4% of patients, respectively. Laboratory test results are shown in Table 2.

Outcomes

We found that 153 patients died (30.2%), 333 were discharged home (65.9%), and 20 (3.9%) remained in the hospital when we finalised data collection on August 27, 2020 (Table 2). A total of 300 of the 506 patients (59.3%) required ICU admission, 227 (44.9%) needed invasive ventilation, 179 (35.4%) needed vasopressors, and 68 (13.4%) required dialysis. Of the 353 survivors 46.7% went through the ICU stay, 18.4% used vasopressors and 5.4% needed haemodialysis. All-cause mortality in patients not admitted to the ICU was 8.7%, and in patients who did not need intubation, it was 9.3%. In patients admitted to the ICU, the mortality was 45%, while it was 56% in the intubated patients. Patients on vasopressors had an all-cause mortality of 63.7%, and it was as high as 72.1% in patients who underwent dialysis. The median time from illness onset to ED evaluation was 8 days, and that from onset to home discharge was 22.1 days. The median length of hospital stay was 11 days (Figure 2).

Comparison between survivors and non-survivors

Survivors were significantly younger than non-survivors (57.4 vs 66.2 years old). Myalgia (45.0% vs 24.8%), sore throat (17.0% vs 9.8%), headache (29.8% vs 15.0%), dysgeusia (28.9% vs 9.8%), and anosmia (24.1% vs 9.2%) were significantly more frequent among survivors. In our univariate analysis low blood pressure, high cardiac rate, and hypoxia were associated with an increased risk of death, ICU admission, or invasive ventilation (Table 1).

There were significantly higher levels of C-reactive protein, lactate dehydrogenase, neutrophils, and troponin T and lower levels of lymphocytes in: non-survivors than that in survivors, in patients admitted to the ICU than that in those not admitted to the ICU, and in intubated patients than that in those who were not intubated.

In this study, the D-dimer levels were high, with 86.7% of patients having levels higher than 500 µg/dL, and with survivors having a median level of 1136 U/L, compared with 1851 U/L in non-survivors. Admission troponin T was available for 314 patients, and in 50% of cases it was elevated. Median cardiac troponin T for survivors was 11 ng/L, while it was 38 ng/L for non-survivors. In survivors, 38.3% had an elevated troponin T level, while it was 76.3% in non-survivors.

Revised Text:

We found that 153 patients died (30.2%), 333 were discharged home (65.9%), and 20 (3.9%) remained in the hospital when we finalised data collection on August 27, 2020 (Table 2). A total of 300 of the 506 patients (59.3%) required ICU admission, 227 (44.9%) needed invasive ventilation, 179 (35.4%) needed vasopressors, and 68 (13.4%) required dialysis. Of the 353 survivors, 165 patients (46.7%) went through the ICU stay, 65 (18.4%) used vasopressors and 19 (5.4%) needed haemodialysis. All-cause mortality in patients not admitted to the ICU was 18 patients (8.7%), and in patients who did not need intubation, it was 26 (9.3%). In patients admitted to the ICU, 135 patients (45%) died, while 127 (56%) died in the intubated patients. There were 114 (63.7%) patients on vasopressors who died, and 49 (72.1%) died from those who underwent dialysis. The median time from illness onset to ED evaluation was 8 days, and that from onset to home discharge was 22.1 days. The median length of hospital stay was 11 days (Figure 2).

Comparison between survivors and non-survivors

Survivors were significantly younger than non-survivors (57.4 vs 66.2 years old). Myalgia (159 patients [45.0%] vs 38 patients [24.8%]), sore throat (60 [17.0%] vs 15 [9.8%]), headache (105 [29.8%] vs 23 [15.0%]), dysgeusia (102 [28.9%] vs 15 [9.8%]), and anosmia (85 [24.1%] vs 14 [9.2%]) were significantly more frequent among survivors. In our univariate analysis low blood pressure, high cardiac rate, and hypoxia were associated with an increased risk of death, ICU admission, or invasive ventilation (Table 1).

There were significantly higher levels of C-reactive protein, lactate dehydrogenase, neutrophils, and troponin T and lower levels of lymphocytes in: non-survivors than that in survivors, in patients admitted to the ICU than that in those not admitted to the ICU, and in intubated patients than that in those who were not intubated.

In this study, the D-dimer levels were high, with 332 (86.7%) patients having levels higher than 500 µg/dL, and with survivors having a median level of 1136 U/L, compared with 1851 U/L in non-survivors. Admission troponin T was available for 314 patients, and in 157 (50%) it was elevated. Median cardiac troponin T for survivors was 11 ng/L, while it was 38 ng/L for non-survivors. In survivors, 83 (38.3%) had an elevated troponin T level, while 74 (76.3%) had an elevated troponin T level in non-survivors.

Comment #9: The description of the multivariable analysis requires some more detail to allow it to be reproducible:

1. How were missing predictor variables handled (I assume those with missing outcomes were excluded from the analysis)? Were missing data assumed to be normal? This may require some more information regarding the recording of predictor variables, perhaps by providing the data collection form as an appendix.

2. How was the relationship between continuous predictor variables and outcome modelled, given that some may have non-linear relationships with outcome?

3. How many predictor variables were included in the analysis? The sample size may be insufficient for a large number of predictor variables, and including too many may lead to over-fitting.

4. Was any analysis undertaken to validate the multivariable model?

In general, I think the multivariable analysis is the weakest part of the study. The descriptive analysis is appropriate and clearly presented, allowing readers to understand the characteristics of your population and compare it to other populations. It is more difficult to draw conclusions from the multivariable analysis. The study may lack power for multivariable analysis, creating risks of important predictors being missed, while the model may be over-fitted if too many predictor variables were included. I would either drop the multivariable analysis from the paper or, if retained, ensure the analysis is clearly described and the limitations acknowledged.

Response #9:

Thank you so much for these observations. As we have stated in comment #1 we performed a new multivariate analysis which has been performed appropriately and is much more robust.

The set of variables used in the multivariate analysis has some variables with missing data, The table below shows the percentage of missing data for each variable used in the multivariate analysis.

| **predictor variables** | **missing (%)** |  | **predictor variables** | **missing (%)** |
| --- | --- | --- | --- | --- |
| **Death date** | 0 |  | **Hemoglobin** | 8 |
| **Age** | 0 |  | **Neutrophyls** | 15 |
| **Sore throat** | 0 |  | **Lymphocytes** | 15 |
| **Headache** | 0 |  | **Creatinine** | 7 |
| **Dysgeusia** | 0 |  | **Urea** | 8 |
| **Anosmia** | 0 |  | **D Dimer** | 24 |
| **Mean arterial pressure** | 14 |  | **C reactive protein** | 13 |
| **O_2_ peripheral saturation** | 5 |  | **AST** | 17 |
| **Type of oxygen supplementation** | 0 |  | **LDH** | 26 |
| **Number of comorbidities** | 0 |  | **Roche HS Troponin T** | 38 |
| **Use of ACE inhibitors** | 6 |  | **Arterial lactate** | 43 |
| **Use of ARB** | 7 |  | **Extension of ground glass on CT** | 25 |

The chained equations algorithm that imputes incomplete multivariate data was applied in the dataset.

2. A generalized linear model (logistic model) was adjusted, with penalized regression (lassos). This model was log transformed to obtain a linear predictor between the responses and the predictors.

3. To solve the problem of over-fitting and insufficient sample size, the binomial family model with lasso regression was used, which is robust when the sample size is reduced compared to the number of predictors in the model.

Some of the predictors can be highly correlated which can inflate the variance. Lasso regression allows for selection of the most suitable predictors because it produces shrinkage estimates with predictive errors potentially lower than the common least squares.

4. For multivariate analysis we considered a class of penalized objective functions which, construct a homogeneous pooled objective function and combine the objective functions for each of the imputed datasets together and jointly optimize the collective objective function[6, 7]

This analysis can overcome the problem of imputation and selection of predictors at the same time, despite the small sample size for the number of predictors, the existence of highly correlated predictors that inflate the variance and the loss of several patients by missing data.

Finally we did not validate the multivariable model, except to check its perfomance in the same dataset from which it was derived.

Comment # 10: Finally, PLOS ONE has reviewed a paper from my research team describing ED attendances with suspected COVID-19 across the UK. You may wish to contrast your findings with ours in your discussion. The pre-print is available here (<https://www.medrxiv.org/content/10.1101/2020.08.10.20171496v1>) and I am happy to share our revised paper, if this is OK with the PLOS ONE editors.

Response #10:

One way to validate the model is done with cross-validation, because the deduction of the predictors was performed on five sets of imputed data making the predictors present a certain stability. Performing a replication of the model in a database completely independent of our current study, would be the optimal situation Perhaps we could colaborate to validate our model in the dataset from the reviewer's ED in a future study.

1.	Meini S, Suardi LR, Busoni M, Roberts AT, Fortini A. Olfactory and gustatory dysfunctions in 100 patients hospitalized for COVID-19: sex differences and recovery time in real-life. Eur Arch Otorhinolaryngol. 2020. doi: 10.1007/s00405-020-06102-8. PubMed PMID: 32500326; PubMed Central PMCID: PMCPMC7271634.

2.	Lapostolle F, Schneider E, Vianu I, Dollet G, Roche B, Berdah J, et al. Clinical features of 1487 COVID-19 patients with outpatient management in the Greater Paris: the COVID-call study. Internal and emergency medicine. 2020. doi: 10.1007/s11739-020-02379-z. PubMed PMID: 32474850; PubMed Central PMCID: PMCPMC7260461.

3.	Tong JY, Wong A, Zhu D, Fastenberg JH, Tham T. The Prevalence of Olfactory and Gustatory Dysfunction in COVID-19 Patients: A Systematic Review and Meta-analysis. Otolaryngol Head Neck Surg. 2020;163(1):3–11. doi: 10.1177/0194599820926473. PubMed PMID: 32369429.

4.	World Health Organization. Clinical management of severe acute respiratory infection when novel coronavirus (2019-nCoV) infection is suspected: interim guidance. . 2020.

5.	Lu X, Wang L, Sakthivel SK, Whitaker B, Murray J, Kamili S, et al. US CDC Real-Time Reverse Transcription PCR Panel for Detection of Severe Acute Respiratory Syndrome Coronavirus 2. Emerg Infect Dis. 2020;26(8). doi: 10.3201/eid2608.201246. PubMed PMID: 32396505.

6.	Du J, Boss J, Han P, Beesley LJ, Goutman SA, Batterman S, et al. Variable selection with multiply-imputed datasets: choosing between stacked and grouped methods. arXiv: Methodology. 2020.

7.	Zahid FM, Faisal S, Heumann C. Variable selection techniques after multiple imputation in high-dimensional data. Statistical Methods & Applications. 2020;29:553-80.
